# Comparing Jupiter’s Equatorial X‐Ray Emissions With Solar X‐Ray Flux Over 19 Years of the Chandra Mission

**DOI:** 10.1029/2022JA030971

**Published:** 2022-12-03

**Authors:** S. C. McEntee, C. M. Jackman, D. M. Weigt, W. R. Dunn, V. Kashyap, R. Kraft, C. K. Louis, G. Branduardi‐Raymont, G. R. Gladstone, P. T. Gallagher

**Affiliations:** ^1^ School of Cosmic Physics DIAS Dunsink Observatory Dublin Institute for Advanced Studies Dublin Ireland; ^2^ School of Physics Trinity College Dublin Dublin Ireland; ^3^ Department of Physics and Astronomy University College London London UK; ^4^ Centre for Planetary Sciences at UCL/Birkbeck London UK; ^5^ Harvard‐Smithsonian Center for Astrophysics Smithsonian Astrophysical Observatory Cambridge MA USA; ^6^ Mullard Space Science Laboratory Department of Space and Climate Physics University College London Dorking UK; ^7^ Space Science and Engineering Division Southwest Research Institute San Antonio TX USA; ^8^ Department of Physics and Astronomy University of Texas at San Antonio San Antonio TX USA

**Keywords:** Jupiter, X‐ray, disk, solar, Chandra, GOES

## Abstract

We present a statistical study of Jupiter’s disk X‐ray emissions using 19 years of Chandra X‐Ray Observatory (CXO) observations. Previous work has suggested that these emissions are consistent with solar X‐rays elastically scattered from Jupiter’s upper atmosphere. We showcase a new pulse invariant (PI) filtering method that minimizes instrumental effects which may produce unphysical trends in photon counts across the nearly two‐decade span of the observations. We compare the CXO results with solar X‐ray flux data from the Geostationary Operational Environmental Satellites X‐ray Sensor for the wavelength band 1–8 Å (long channel), to quantify the correlation between solar activity and Jovian disk counts. We find a statistically significant Pearson’s Correlation Coefficient of 0.9, which confirms that emitted Jovian disk X‐rays are predominantly governed by solar activity. We also utilize the high spatial resolution of the High Resolution Camera Instrument on‐board the CXO to map the disk photons to their positions on Jupiter’s surface. Voronoi tessellation diagrams were constructed with the Juno Reference Model through Perijove 9 internal field model overlaid to identify any spatial preference of equatorial photons. After accounting for area and scattering across the curved surface of the planet, we find a preference of Jovian disk emission at 2–3.5 Gauss surface magnetic field strength. This suggests that a portion of the disk X‐rays may be linked to processes other than solar scattering: the spatial preference associated with magnetic field strength may imply increased precipitation from the radiation belts, as previously postulated.

## Key Points

- We present a statistical study of Jovian disk X‐rays from 19 years worth of Chandra data showing a strong correlation with solar X‐ray flux
- Jovian disk emissions are predominantly governed by solar activity. Pearson’s Correlation Coefficient of 0.9 found between the data
- Analysis of spatial morphology of the disk emissions reveals preference of disk emission at 2–3.5 Gauss magnetic field strength

## 1 Introduction

X‐ray emissions from Jupiter have been observed since 1979, and were first detected by the imaging proportional counter and high resolution imaging detectors on the Einstein Observatory (Metzger et al., 1983), with the emissions characterized into the auroral (high‐latitude) regions and the planetary disk (low‐to mid‐latitude). Differing driving mechanisms have been suggested to explain the properties of the X‐rays emitted from these regions. Several studies have reported the strong conclusion that the X‐rays emitted from Jupiter’s planetary disk are likely to be correlated with solar X‐rays, with spikes/peaks in the Jovian light curve coincident with light travel‐time‐corrected Jupiter‐facing solar flares (e.g., Bhardwaj et al., 2005, 2006; Branduardi‐Raymont et al., 2007, 2010; Cravens et al., 2006; Dunn et al., 2020a; Elsner, Lugaz, et al., 2005; Elsner, Ramsay, et al., 2005). This interpretation is based on data taken from the Chandra X‐ray Observatory (CXO) (Weisskopf et al., 2000) and the X‐ray Multi‐Mirror Mission (XMM‐Newton) (Jansen et al., 2001), fitted with the EUV97 solar proxy model (Tobiska & Eparvier, 1998), that suggest the vast majority (∼90%) of disk X‐ray emissions are produced from solar X‐rays elastically scattered from Jupiter’s upper atmosphere, with ∼10% fluorescent production of carbon K‐shell X‐rays from methane (Cravens et al., 2006; Maurellis et al., 2000). Previous case studies have reported instances where the disk X‐rays show similar day‐to‐day variability as the solar X‐rays (Bhardwaj et al., 2005), with no evidence of the quasi‐periodic flaring occasionally seen in the auroral X‐rays (e.g., Gladstone et al., 2002; Jackman et al., 2018; Weigt, Jackman, et al., 2021).

Branduardi‐Raymont et al. (2010) found an apparent correlation between solar X‐ray flux and disk X‐ray power for both Jupiter and Saturn for a variety of observations. Furthermore, the equatorial spectrum of Jupiter during solar maximum is best fitted by coronal spectral models with temperatures in the energy range 0.4–0.5 keV, with additional line emission from lines commonly seen in the solar X‐ray spectrum at maximum activity and during flares, such as Mg XI (1.35 keV) and Si XIII (1.86 keV) (Branduardi‐Raymont et al., 2007). Dunn et al. (2020a) states that the peak of the spectrum shifts to lower energies during the solar declining phase (0.29 ± 0.02 keV) and solar minimum (0.18 ± 0.02 keV). This combination of spectral and temporal analysis, albeit from a small selection of case study events, has given further credence to the interpretation that the disk and auroral X‐rays are produced by different processes. The auroral emissions can be split into hard X‐rays (>2 keV), which result from X‐ray bremsstrahlung, and soft X‐rays (<2 keV), which are likely produced by charge exchange between precipitating ions and neutrals in the Jovian atmosphere (Branduardi‐Raymont et al., 2008; Elsner, Lugaz, et al., 2005; Elsner, Ramsay, et al., 2005; Houston et al., 2020). Alternatively, Jovian disk X‐ray emission is thought to be produced predominantly by scattering of solar X‐rays in the Jovian upper atmosphere.

One other interesting property of the Jovian disk emission is the observation of a small but statistically significant hour angle dependence in disk count rate and possible link to surface magnetic field strength, with higher X‐ray intensity in regions of low surface magnetic field strength (Elsner, Lugaz, et al., 2005; Elsner, Ramsay, et al., 2005; Gladstone et al., 1998; Waite et al., 1997). This preferential emission of equatorial X‐rays from regions of low surface magnetic field can be explained by assuming these regions allow a larger atmospheric loss cone, which enables the precipitation of otherwise trapped ions and electrons from the radiation belts directly into the upper atmosphere, where they undergo charge–exchange interactions to produce X‐rays (Waite et al., 1997). Indeed, Juno observations (Bolton et al., 2017) of the radiation belts show regions of low surface field strength where otherwise trapped populations are lost to the atmosphere (Kollmann et al., 2021). Examination of the infrared (IR) emissions also showed a link to planetary magnetic field strength (Stallard et al., 2018), perhaps due to horizontal fields inhibiting the precipitation of H_2_ into the atmosphere. Very recently, high resolution magnetic field data from the Juno spacecraft have revealed a region of intense localized magnetic field near Jupiter’s equator (Moore et al., 2018). Now that we have a highly resolved map of Jupiter’s magnetic field thanks to Juno, as well as high spatial resolution X‐ray measurements from CXO, we can make an analogous statistical map of the X‐rays on the planet and quantitatively explore the links.

Since the earlier works on Jovian disk X‐rays, there have been a wealth of new observations of Jupiter, scheduled to coincide with the in situ exploration by the NASA Juno spacecraft. The motivation for this study is to examine the complete catalog of high spatial resolution Chandra observations (up to and including 2019) to quantify any correlation with solar X‐ray flux, and to probe the distribution of photons across Jupiter. We will then investigate any significant clustering of emission in the context of local magnetic anomalies or other dynamic processes at Jupiter. This is the first study of its kind to explore this over ∼ two full solar cycles using the Chandra catalog. The goals of this work include: (a) tracking Jovian X‐rays from the planetary disk as a function of solar cycle, (b) exploring the extent to which these disk X‐rays correlate to solar X‐ray activity, (c) quantifying the spread of X‐ray emission across the disk.

In this study, we utilize Jovian X‐ray data taken over 19 years with the CXO’s High Resolution Camera (HRC) and compare them to corresponding solar X‐ray flux data from the Geostationary Operational Environmental Satellites (GOES). Section 2 of this paper details the data set used in this study, and the processing methods that were employed to ensure consistency across the time span of the data. Section 3 shows results from temporal and spatial analyses of the data set, and Section 4 offers an interpretation of the results and poses questions for future investigation.

## 2 Data Sets and Methods

### 2.1 Data Sets

The CXO conducted observations of Jupiter 29 times between 2000 and 2019 using the on‐board High‐Resolution Camera Imaging (HRC‐I) instrument. The HRC‐I contains a single large‐format microchannel plate, providing high spatial resolution of ∼0.4 arcsec over a 30 arcmin × 30 arcmin field of view. The best image quality is found in the center of the field of view, where the camera’s aim point is located. This allows for the X‐ray time‐tagged photons to be mapped to their specific location on Jupiter’s surface in System III (SIII) latitude and SIII longitude (where System III is a left‐handed co‐ordinate system which rotates with the planet, and where the *z*‐axis is defined by the spin axis of Jupiter). HRC‐I is, however, limited by its poor energy resolution, rendering it unable to distinguish between hard and soft X‐rays. Of this data set, 21 observations have taken place since 2016 (starting with Juno’s approach and arrival at Jupiter). Many of the Juno‐era observations were taken near the perijoves of the Juno spacecraft, with a few timed to coincide with apojove, or with other key magnetospheric encounters (such as current sheet crossings). The remaining observations coincided with campaigns in other wavelenghts (including hubble space telescope (HST) UV auroral observations). Table 1 shows key descriptors of each of the Chandra HRC‐I observations. The Earth–Jupiter (E–J) distances, Sun–Jupiter (S–J) distances, Sun–Earth (S–E) distances, and Earth–Sun–Jupiter (E–S–J) angles were all obtained using the JPL Horizons program (data available at https://ssd.jpl.nasa.gov/horizons/app.html%23/).

**Table 1:** List of Chandra HRC‐I Observations of Jupiter From 2000 to 2019, Including the Observation ID (ObsID), Exposure Time, Disk Counts, Net Disk Count Rate, Earth–Jupiter (E–J) Distance, Sun–Jupiter (S–J) Distance, Sun–Earth (S–E) Distance and Earth–Sun–Jupiter (E–S–J) Angle

ObsID	Start date (year–month–day hr:min:s)	Exp time (ks)	Disk counts	Net disk count rate (Cts/ks/px^2^)	E–J distance (AU)	S–J distance (AU)	S–E distance (AU)	E–S–J angle (deg)
^a^1862	2000–12–18 09:54:27	37	1,840	4.69 × 10^−4^	4.13	5.04	0.98	19.05
^b^2519	2003–02–25 00:22:24	72	1,498	1.35 × 10^−4^	4.41	5.32	0.99	21.48
^a^15,669	2014–04–15 20:44:11	40	926	2.32 × 10^−4^	5.37	5.23	1.00	92.29
^a^15,670	2014–04–20 02:20:37	42	756	0.81 × 10^−4^	5.43	5.23	1.01	96.08
^b^15,671	2014–04–08 08:19:16	43	1,001	2.21 × 10^−4^	5.25	5.23	1.00	85.55
^b^15,672	2014–04–12 22:10:37	42	876	1.89 × 10^−4^	5.32	5.23	1.00	89.66
^a^16,299	2014–04–10 01:10:29	40	991	2.61 × 10^−4^	5.27	5.23	1.00	87.06
^a^16,300	2014–04–17 12:20:38	42	937	2.17 × 10^−4^	5.39	5.23	1.00	93.78
^b^18,301	2017–02–02 09:58:06	35	937	−0.01 × 10^−4^	5.03	5.46	0.99	59.45
^b^18,302	2017–05–19 00:28:41	43	1,404	0.18 × 10^−4^	4.69	5.46	1.01	37.12
^a^18,608	2016–05–24 10:23:06	42	967	0.25 × 10^−4^	5.18	5.44	1.01	69.74
^b^18,609	2016–06–01 11:32:08	42	914	0.14 × 10^−4^	5.30	5.44	1.01	76.85
18,676	2017–03–27 08:32:05	11	326	0.02 × 10^−4^	4.48	5.46	1.00	10.53
^b^18,677	2017–07–10 21:12:27	42	1,081	0.25 × 10^−4^	5.43	5.45	1.02	83.66
^b^18,678	2018–03–31 23:11:51	41	1311	0.15 × 10^−4^	4.62	5.42	1.00	34.05
^b^18,679	2018–05–24 00:00:53	42	1,457	0.15 × 10^−4^	4.43	5.41	1.01	13.55
^b^18,680	2018–09–06 20:39:56	43	895	0.17 × 10^−4^	5.75	5.38	1.01	106.74
^b^20,000	2017–02–28 12:40:03	74	2,422	0.09 × 10^−4^	4.68	5.46	1.00	34.86
^b^20,001	2017–06–18 18:39:05	39	1,063	0.12 × 10^−4^	5.09	5.45	1.02	64.23
^b^20,002	2017–08–06 01:56:57	38	757	−0.05 × 10^−4^	5.82	5.45	1.01	106.67
^b^20,733	2018–04–01 10:39:09	41	1,264	−0.04 × 10^−4^	4.62	5.42	1.00	33.62
22,146	2019–07–13 01:27:31	27	865	0.12 × 10^−4^	4.42	5.29	1.02	28.47
22,147	2019–07–13 21:09:39	25	798	0.13 × 10^−4^	4.43	5.29	1.02	29.18
22,148	2019–07–15 13:00:05	27	827	−0.01 × 10^−4^	4.44	5.29	1.02	30.64
22,149	2019–07–16 08:45:59	27	941	0.53 × 10^−4^	4.45	5.29	1.02	31.36
22,150	2019–07–18 20:19:01	27	904	0.40 × 10^−4^	4.48	5.29	1.02	33.52
22,151	2019–09–08 22:59:25	27	705	0.39 × 10^−4^	5.18	5.27	1.01	79.40
^b^22,159	2019–05–29 03:27:35	38	1,305	0.07 × 10^−4^	4.31	5.30	1.01	10.79

*Note*. One observation (ObsID 18303) is omitted from the data set due to Jupiter’s position on the chip of the detector being shifted away from the aim point.

^a^ Relative high solar activity cases (as defined in Section 3.1).

^b^ Relative low solar activity cases.

**Figure 1:**
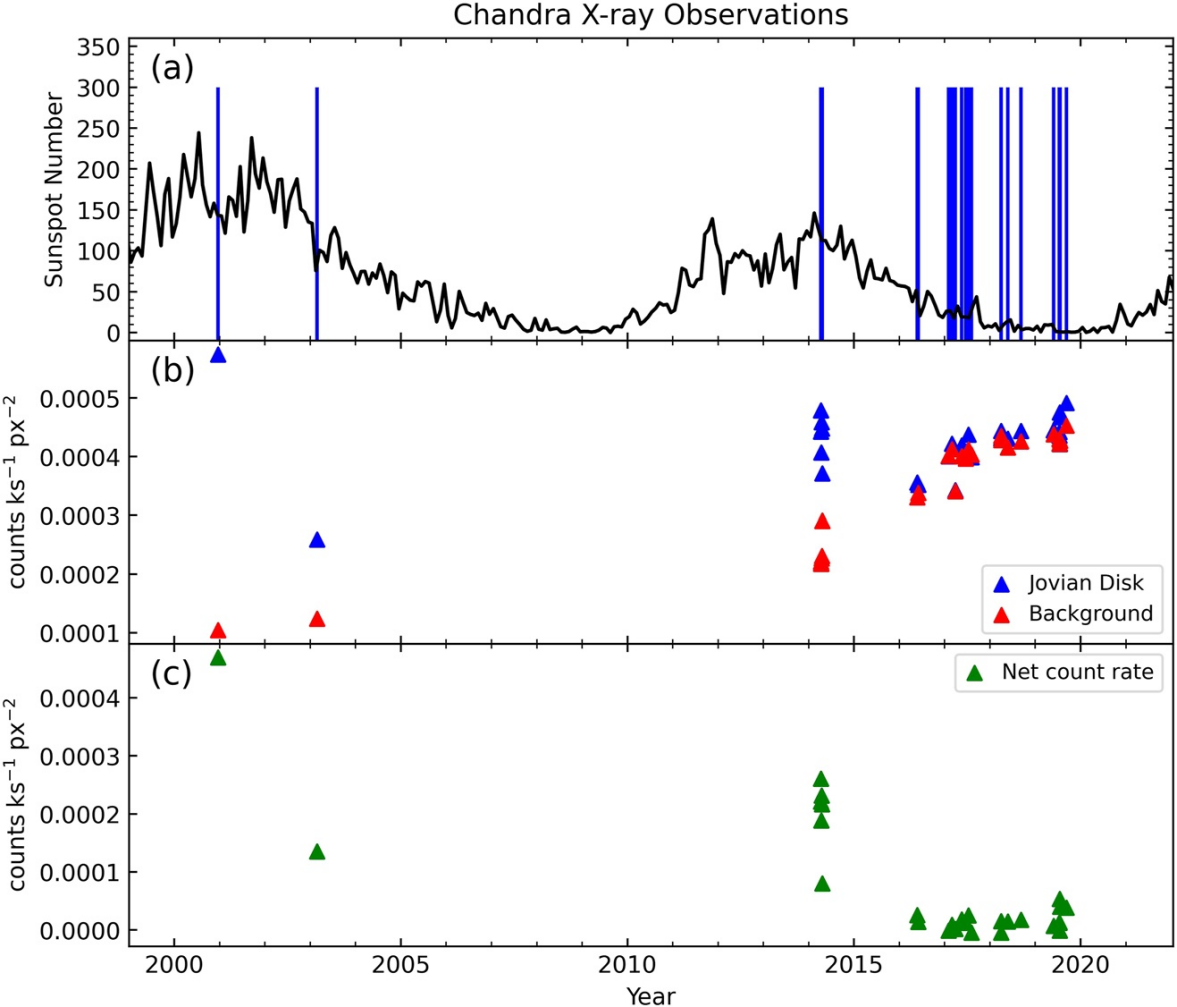
Chandra X‐ray observations (CXO) of Jupiter from 2000 to 2019. (a) Monthly sunspot number (from SIDC https://www.sidc.be/silso/home) with times of CXO observations overlaid by vertical lines. (b) Jovian disk (blue) and background (red) count rates per unit area (cts ks^−1^ px^−2^) for each HRC‐I observation of Jupiter. (c) Net count rate (green) of Jovian disk after subtracting the background (cts ks^−1^ px^−2^). Disk region defined as latitudes from −55° to +45° SIII lat.

We use the same 29 observations which were explored in a statistical study by Weigt, Jackman, et al. (2021) of the northern auroral emissions. Like that study, one observation is omitted (ObsID 18303) due to Jupiter’s position on the chip of the detector being shifted away from the aim point. The result of this misalignment was that the mapping procedure could not be performed accurately as the point spread function (PSF) increases with distance from the center of the detector, leading to large uncertainties. The 28 HRC‐I observation times are displayed in panel (a) of Figure 1 (blue vertical lines) overplotted to the smoothed monthly sunspot number to give an indication of the coverage of Jovian X‐ray observations with solar cycle. The calculation of the count rates which appear in panels (b) and (c) will be discussed in detail in Section 2.2.3.

In addition to Chandra data, we utilize data from GOES, a constellation of satellites which orbit the Earth in a geosynchronous orbit, with near‐constant viewing of the Sun. The X‐ray Sensor (XRS) on‐board has the ability to monitor changes in solar X‐ray flux with 3‐s time resolution over the corresponding time window of HRC‐I observations of Jupiter. GOES XRS provides solar X‐ray fluxes for the wavelength band 1–8 Å (long channel), corresponding to an energy range 1.55–12.4 keV. In comparison, Jupiter’s disk emissions during solar maximum are dominated by Fe‐lines that peak at 0.7–0.9 keV, with further contributions from Ne and Mg up to 1.5 keV. However, the peak of this spectrum shifts to lower energies during the solar declining phase and solar minimum (Dunn et al., 2020a). This discrepancy between the energy ranges of the respective satellites means that the lower limit of the XRS energy range will exceed the peak of the Jovian disk X‐ray brightness. Unfortunately, there are no solar X‐ray data available in the 0.1–1.5 keV range to compare with the Jovian disk X‐rays.

For this study, data were consistently available from the G10 satellite for CXO observations prior to 2011, and the G15 satellite supplied data for all observations over the period 2014–2019. The data from these satellites, and the comparison with the corresponding Jovian disk light‐curves, are analyzed in Section 3.1.

In order to be able to quantitatively compare the number of Jovian disk counts obtained for observations spanning this 19‐year interval, it is critical that the processing pipeline takes account of any instrumental changes over time. For this, we needed to develop a new filtering method for the Jovian X‐ray photons and the off‐Jupiter X‐ray background, discussed below in Section 2.2.

### 2.2 Methods

#### 2.2.1 Updates to Mapping Algorithm and Photon Selection Pipeline

**Figure 2:**
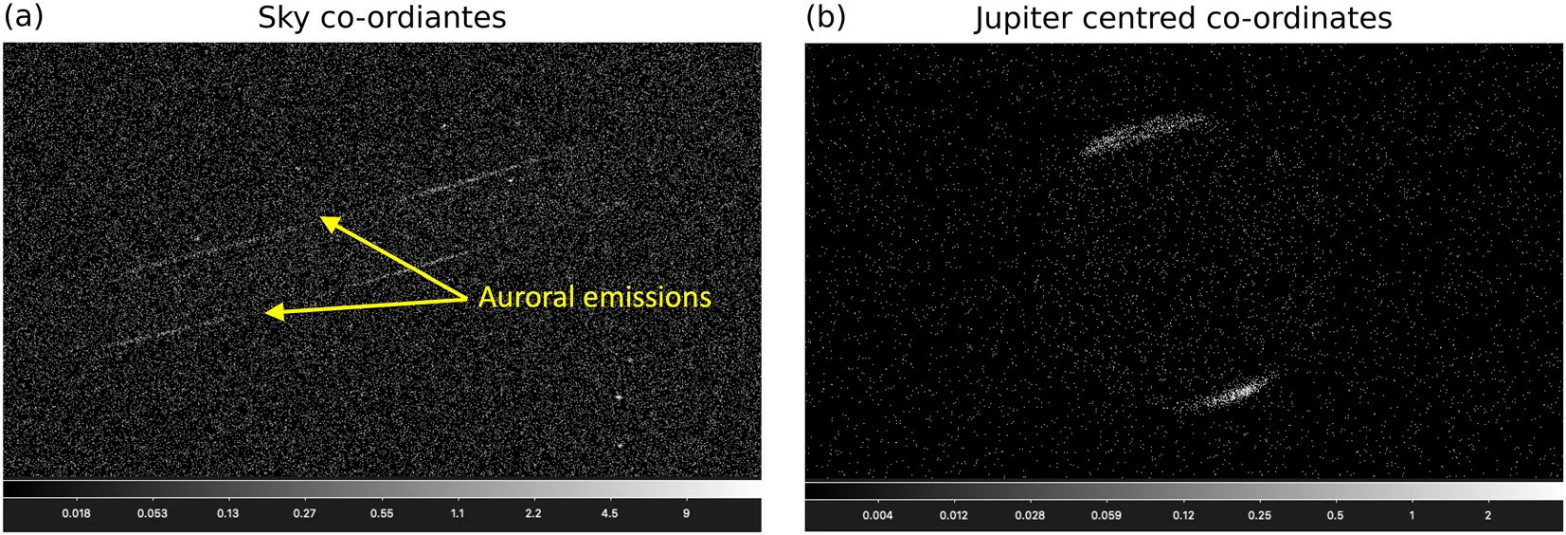
Chandra HRC‐I output files in (a) sky co‐ordinates and (b) Jupiter centered co‐ordinates. Transformation between (a) and (b) was done using the SSO_FREEZE algorithm. Colourbars are in units of counts.

The raw data obtained from HRC‐I first have to be transformed into a frame of reference centered on Jupiter. This is done using the SSO_FREEZE algorithm (see https://cxc.cfa.harvard.edu/ciao/ahelp/sso_freeze.html), which uses appropriate ephemerides data from the JPL Horizons program and Chandra orbit ancillary data from the Chandra X‐ray Center to account for Jupiter’s motion on the sky and the relative position of the detector. The raw data are reprojected from sky *x* and *y* co‐ordinates to a reference frame which is fixed to the motion of Jupiter. This helps to eliminate the “blurring” seen in the sky *x* and *y* co‐ordinates. Figure 2 shows the output images in both (a) sky co‐ordinates and (b) Jupiter centered co‐ordinates. The streaks in (a) display the motion of Jupiter’s northern and southern aurorae across the detector.

**Figure 3:**
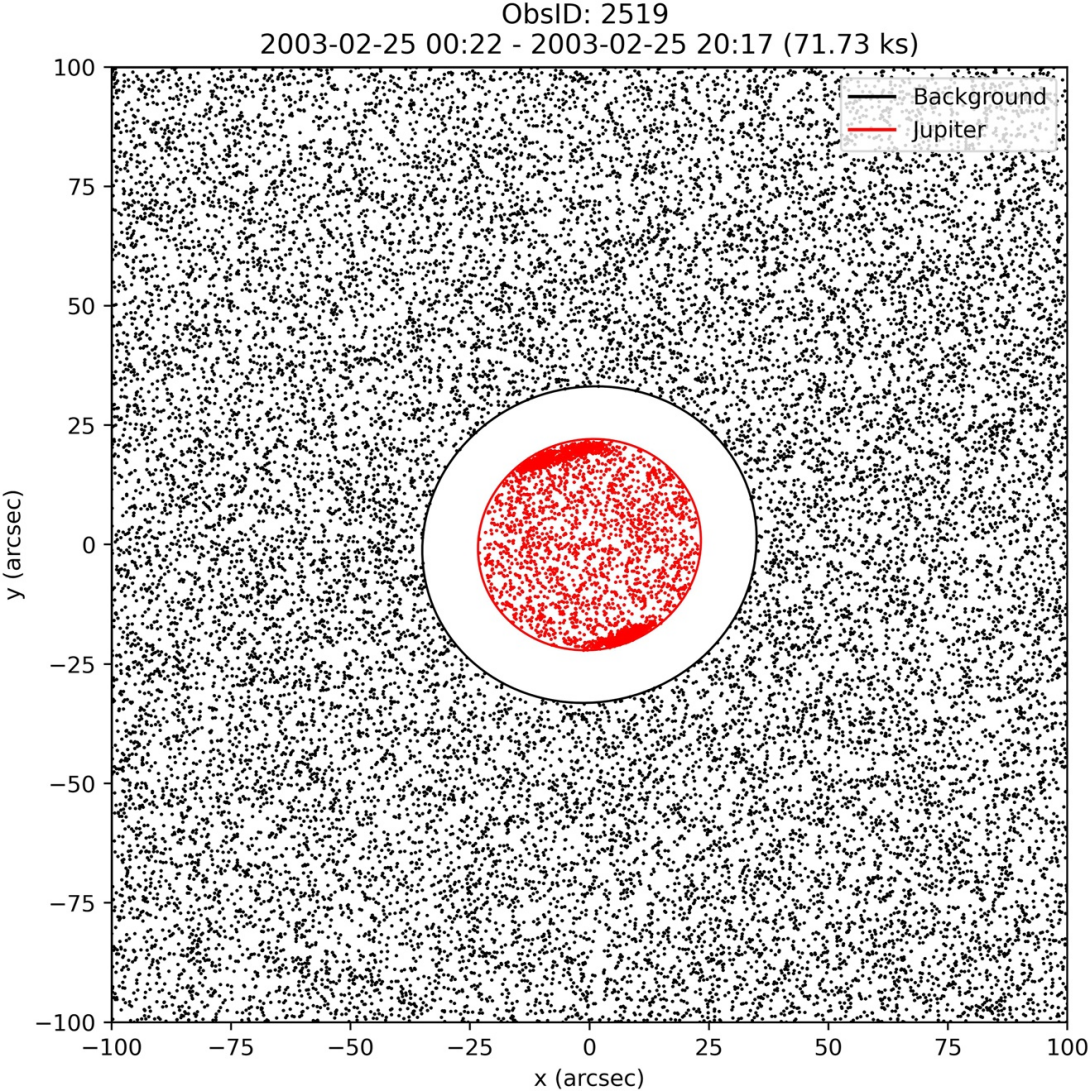
Example of the typical output of the GO_CHANDRA photon selection algorithm, showing the ellipse region (red) constrained to the limb of the planet. Background photons are shown in black. Photons that lie neither on Jupiter nor within the background region have not been included, so the space between these regions is completely empty.

Once completed, a separate GO_CHANDRA algorithm (Gladstone et al., 2002) is employed to map the time‐tagged photons to their respective positions on Jupiter’s surface, which enables the selection of photons according to their positions and times. In previous versions of this algorithm used in published works (Gladstone et al., 2002; Jackman et al., 2018; Weigt et al., 2020), Jupiter was centered on the center of the chip on HRC‐I, and a circle of radius 30 arcsec was drawn around the planet, including all photons within this region. This approximation used by the algorithm is sufficient when looking at the more intense auroral emissions at the poles (e.g., Elsner, Lugaz, et al., 2005; Elsner, Ramsay, et al., 2005; Gladstone et al., 1998). When analyzing the disk emissions however, we want to ensure that we reduce the contamination from X‐rays outside of Jupiter’s disk. As a result, we have produced a new update to this photon selection procedure, which incorporates the ellipticity of Jupiter, and generates a tilted ellipse based on the tilt angle of Jupiter’s north pole and the planet’s angular diameter (both quantities obtained via JPL Horizons). Photons are selected on the basis of whether they lie within this ellipse region. The result is that this updated method better constrains the limb of Jupiter, thus removing a significant proportion of sky background counts located near the limb of the planet that were previously included as Jovian photons (see Figure 3). This data processing pipeline is provided at https://doi.org/10.5281/zenodo.5657141 (Weigt et al., 2022).

#### 2.2.2 Pulse Invariant Filtering Method

Maintaining consistency is crucial when compiling a data set over such a large time span, particularly when using quantitative information such as total photon counts to infer information about the level of Jovian activity and solar driving. Over time, instrument degradation becomes a key factor on‐board CXO. In the case of HRC‐I, the gain of the instrument has been decreasing over time (see https://cxc.cfa.harvard.edu/cal/Hrc/Documents/Gain/hrci_sampgain_nov2009.pdf), and the calibration team at Chandra have developed procedures to measure this trend (Posson‐Brown & Kashyap, 2007). Originally, the nominal gain metric for HRC was the Pulse Height Amplitude (PHA), which gave the sum of all detector amplifier signals. Another quantity, the scaled sum of amplifier signals (SUMAMPS), gives the sum of signals from the three amplifiers nearest the X‐ray photon signal on each axis. For HRC‐I, the PHA values are limited by saturation at PHA = 255, while SUMAMPS are not, thus providing a better capability for spectral discrimination. Due to the superiority of SUMAMPS for gain measurements on HRC, scaled SUMAMPS (SAMP) has become the standard gain measure. The spatial variance of SAMP is much less than for PHA, and it also has the advantage of not being integerised. The scaling is done by the amplifier scale factor values (*AMP*_*SF*) as follows:

(1) $$SAMP=\frac{SUMAMPS\times 2^{AMP\text{\_}SF-1}}{C},$$

where C is a constant. For HRC‐I, *C* = 148, and this value is chosen so that the SAMP and PHA distributions match closely. The SAMP and PHA distributions shift to lower channels over time as the gain of HRC‐I decreases. To account for this, another quantity, called Pulse Invariant (PI), is introduced. This quantity removes the time‐dependence of the SAMP values, creating a distribution similar to that produced at the beginning of the Chandra mission. SAMP can be converted to PI using a multiplicative gain correction factor, g:

(2) PI=g×SAMP,

where

$$\begin{array}{lll} g & = & 1.0418475+0.020125799\;\left(Y-2000\right)+0.010877227\;{\left(Y-2000\right)}^{2}\;\\ & & -\;0.0014310146\;{\left(Y-2000\right)}^{3}+\left(5.8426766\times 10^{-5}\right)\;{\left(Y-2000\right)}^{4}, \end{array}$$

where *Y* is the start of the observation in decimal year.

After the PI has been calculated for each X‐ray photon detected, we then apply a filter to the source (Jupiter) PI spectrum whereby we only include photons if they lie in the PI channel range 10–250. This channel range was selected to contain the region of the PI spectrum where the source dominates the background. The corresponding PI range is then applied to our X‐ray background spectrum, removing a larger (in general) percentage of background than that removed from the source. Our background region is defined as the area outside an ellipse 1.5 times the size of Jupiter, and inside a square region of length 200 arcsec, shown in Figure 3 as the black region. Photons that do not emanate from Jupiter and are not contained within the background region are not included in Figure 3, resulting in the white space that is observed between the boundaries of the planet (red) and background (black) regions.

This important additional step ensures that we remove as much background contamination from our Jupiter region and should be applied to archival and upcoming Chandra data. We note that these effects occur with observations of other planets such as Saturn (Weigt, Dunn, et al., 2021) and Uranus (Dunn et al., 2021) and should be applied to all CXO HRC‐I planetary observations.

#### 2.2.3 Disk Region Selection

**Figure 4:**
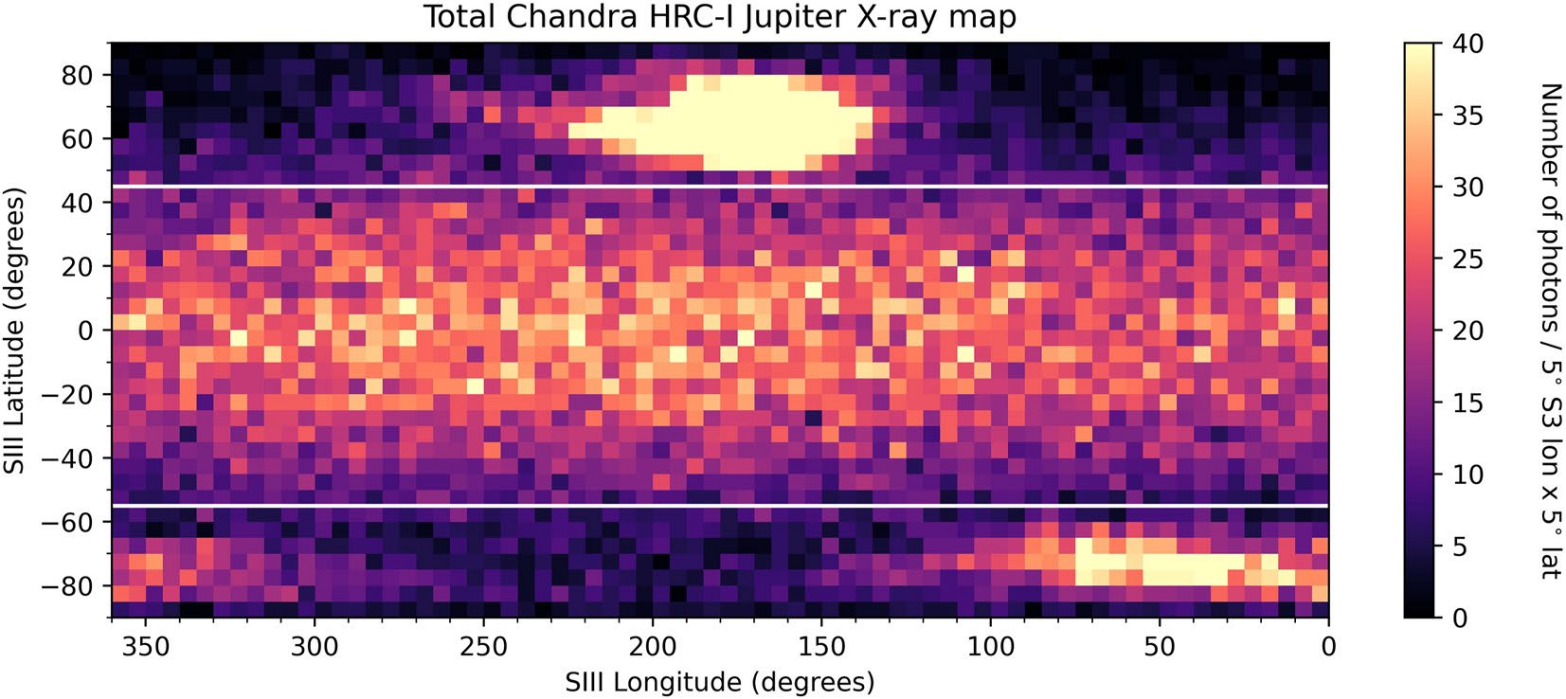
X‐ray heatmap containing the photons detected from all 28 HRC‐I observations of Jupiter from 2000 to 2019. Photons are binned into 5° SIII longitude × 5° SIII latitude bins and the colourbar is saturated at 40 photons per bin to display structure on the disk. White horizontal lines represent the latitude boundaries of the disk region, which are 45° SIII latitude in the north and −55° SIII latitude in the south.

In order to obtain the disk count rates for each of the 28 HRC‐I observations of Jupiter that are displayed in panel (b) of Figure 1, we first must define the boundaries of the Jovian disk. This constraint on latitude was defined using the total X‐ray map illustrated in Figure 4. This figure contains the summed total of all Jovian photons across the entire data set, binned into 5° SIII longitude × 5° SIII latitude bins. The colourbar was also saturated at 40 counts/bin to visualize structure on the disk. The boundaries were imposed at latitudes where there was a clear end to the northern and southern aurorae. These boundaries were found to be +45° SIII latitude in the north and −55° SIII latitude in the south. We utilize the method presented in Bhardwaj et al. (2006) by fitting a rectangular box over color‐coded two‐dimensional histograms of Chandra data to isolate the planetary disk region (see Figure 1). Our boundaries take into account the statistical picture over 19 years and reflect a desire to be conservative so as to not erroneously include auroral photons, but also to encompass as much of the disk as possible. Therefore, Figure 4 can be split into three different regions:

- Northern Auroral Region (>+45° SIII latitude),
- Southern Auroral Region (<−55° SIII latitude),
- Planetary Disk Region (−55° to +45° SIII latitude).

This latitude constraint was used to determine the Jovian disk count rates for the HRC‐I observations that are included in Figure 1b. The Jovian disk count rate, along with that of the background region (as defined in Figure 3), were then divided by the areas of the respective regions (in units of px^2^) to give the final count rates that appear in Figure 1b. Finally, the background was subtracted from the Jovian disk count rate to yield the net disk count rates seen in Figure 1c. These net disk count rates are also displayed in Table 1.

#### 2.2.4 Voronoi Tessellation Algorithm

As discussed earlier, the high spatial resolution of HRC enables the mapping of time‐tagged Jovian photons that strike the detector back to their specific location on Jupiter’s surface in SIII longitude and SIII latitude. The spatial morphology of these photons can then be investigated in further detail. For this purpose, we employ Voronoi tessellation diagrams, based on the VOISE (VOronoi Image SEgmentation) algorithm (Guio & Achilleos, 2009). In this method, each photon, or “seed,” has a given location in SIII longitude and SIII latitude. A polygon is then drawn around each seed, enclosing the area that is closer to that seed than any other. Therefore, there are the same number of polygons in the grid as seeds/photons. The result is a spatial map where the concentration of photons can be quantified and compared between different observations by calculating the areas of the polygons (deg^2^). Observations which contain a large number of photons will produce a Voronoi tessellation diagram containing many low‐area polygons. Conversely, a more sparse data set will result in a spatial map containing large polygons of high area. This method was chosen for the spatial analysis in this study as it provides a more automated and objective method of investigating the spatial morphology of the Jovian disk X‐ray photons.

## 3 Results and Discussion

We wish to examine the temporal and spatial properties of Jupiter’s disk emissions in order to move toward a quantitative understanding of their generation mechanism.

### 3.1 Sun–Jupiter X‐Ray Connection

**Figure 5:**
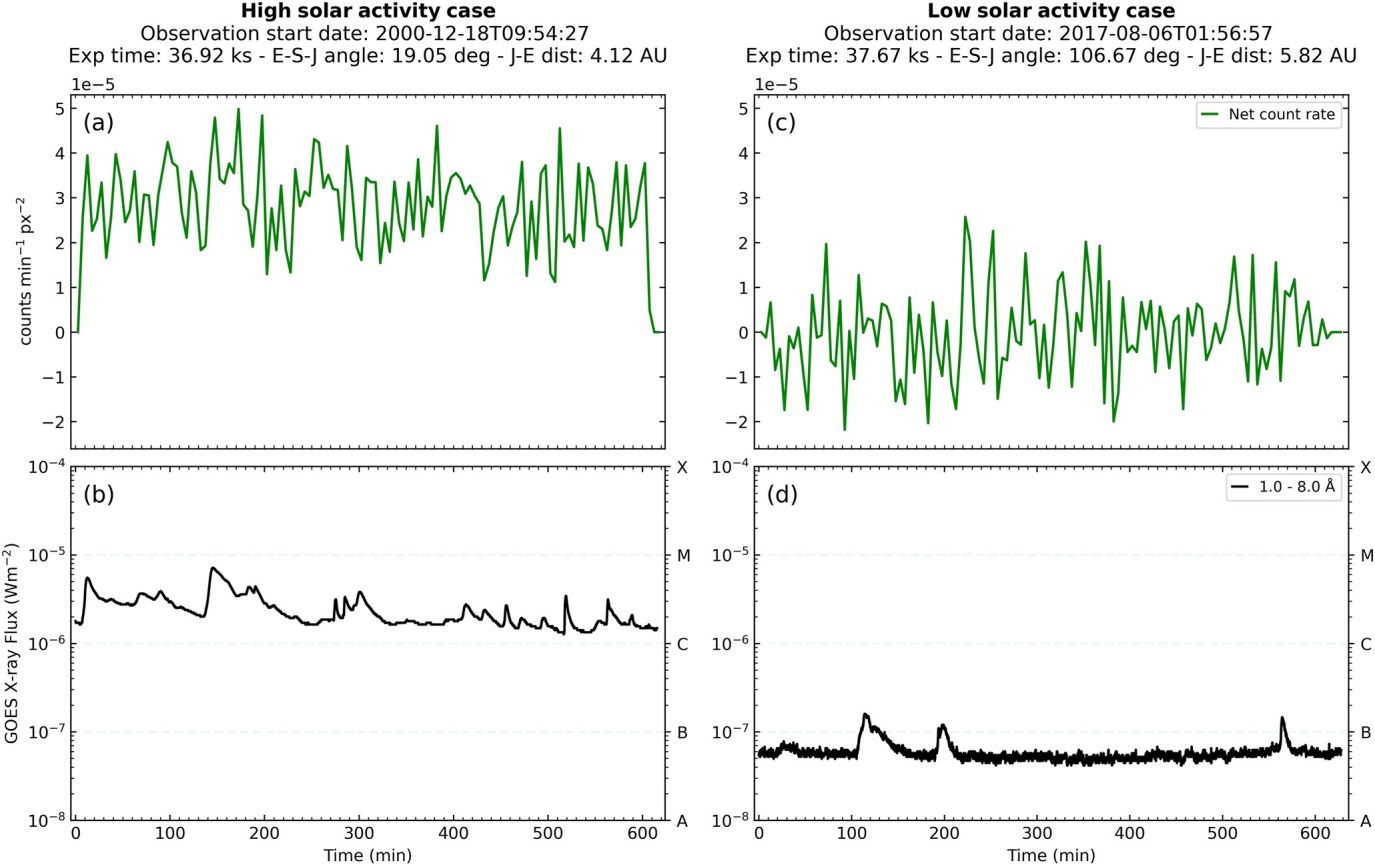
Comparison between HRC‐I Jovian disk net light‐curve (energy range 0.06–10 keV) and GOES solar X‐ray flux (long channel, wavelength band 1–8 Å) for observations that coincided with (a), (b) relatively high (ObsID 1862, 18 December 2000) and (c), (d) low solar activity (ObsID 20002, 6 August 2017). Jovian disk light‐curves are shown in 5‐min bins. The CXO data are shifted backwards in time by the time difference between Sun–Jupiter–Earth and Sun–Earth light travel times. The raw GOES data are used with 3‐s time resolution.

Figure 5 shows comparisons of the net Jovian disk (CXO) and the solar X‐ray (GOES XRS) light curves. Two examples are shown, coinciding with relatively high (a, b) and low (c, d) solar activity. The levels of solar activity were determined on the basis of whether the peak GOES X‐ray flux over the observation window exceeded a quantitative threshold of 10^−6^ Wm^−2^. This threshold was chosen as it represents the mean of our data set, with the peak flux ranging from 2.3 × 10^−8^ to 7.2 × 10^−6^ Wm^−2^. The cases used in this comparison were chosen because they represent the extremes of our data set in terms of Jupiter‐Earth (J‐E) distance (4.13 vs. 5.82 AU). Furthermore, both observations have an exposure time approximately equal to one full Jovian rotation. Panels (a) and (c) of Figure 5 show the net count rates per unit area of Jupiter’s disk region (cts min^−1^ px^−2^) after subtracting the associated particle background for each observation, with the data binned into 5‐min bins. The CXO data are shifted backwards in time by the time difference between Sun‐Jupiter‐Earth and Sun‐Earth light travel times. In this way, we are directly comparing the solar flux observed by GOES to the Jovian disk photons that are detected by HRC‐I on‐board the CXO. The raw GOES data are included with 3‐s time resolution.

The GOES solar X‐ray flux light curves show a greater than order of magnitude change in the baseline flux between the high solar activity case (Figure 5b) and the low solar activity case (Figure 5d). This change is accompanied by a similar change in the Jovian disk net light curve per unit area, indicating that this increase in the net count rate is influenced by the increase in solar X‐ray flux. In order to quantify this link, we must extrapolate this finding to include our entire data set.

**Figure 6:**
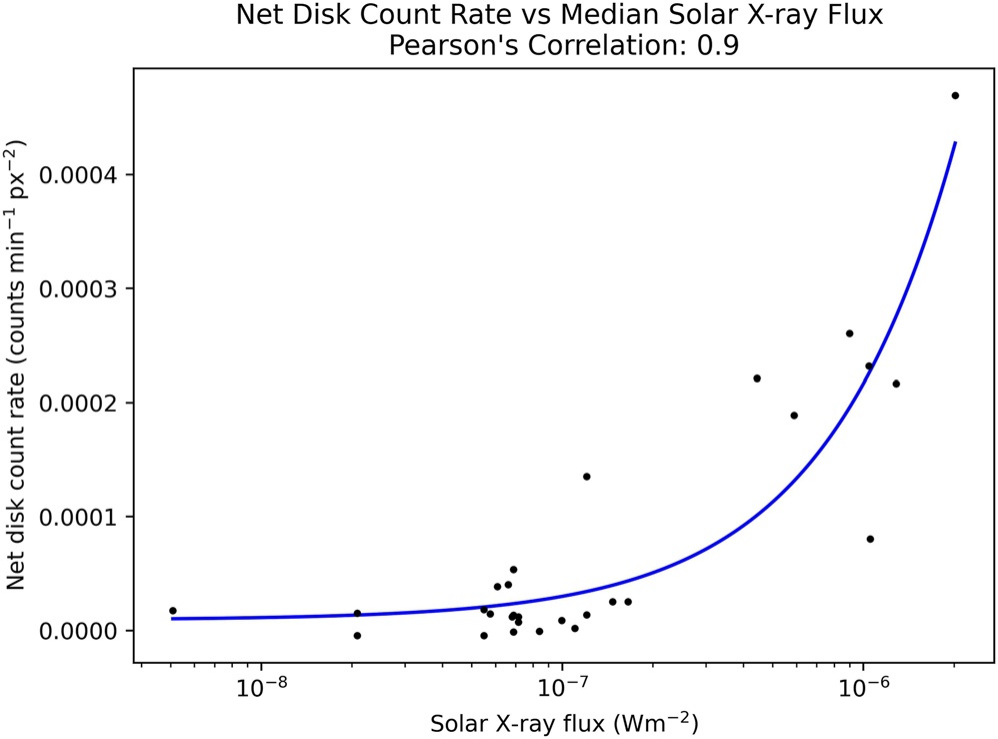
Correlation plot between net Jovian disk count rates per unit area (cts min^−1^ px^−2^) and median GOES solar X‐ray flux (Wm^−2^) over the same observation window. Data are plotted on a linear versus log scale. The linear least squares fit of the data is displayed in blue.

Figure 6 shows the net count rate (cts min^−1^ px^−2^) of the Jovian disk region for each of the 28 HRC‐I observations of Jupiter, plotted against the median GOES solar X‐ray flux (Wm^−2^) over the corresponding observation window. The data sets are found to be in good agreement, with a Pearson’s Correlation Coefficient (PCC) of 0.9. This provides clear evidence that the vast majority of the X‐ray emissions emanating from Jupiter’s planetary disk region are indeed consistent with solar X‐rays elastically scattered from the planet’s upper atmosphere.

### 3.2 Spatial Morphology of Jovian Disk

**Figure 7:**
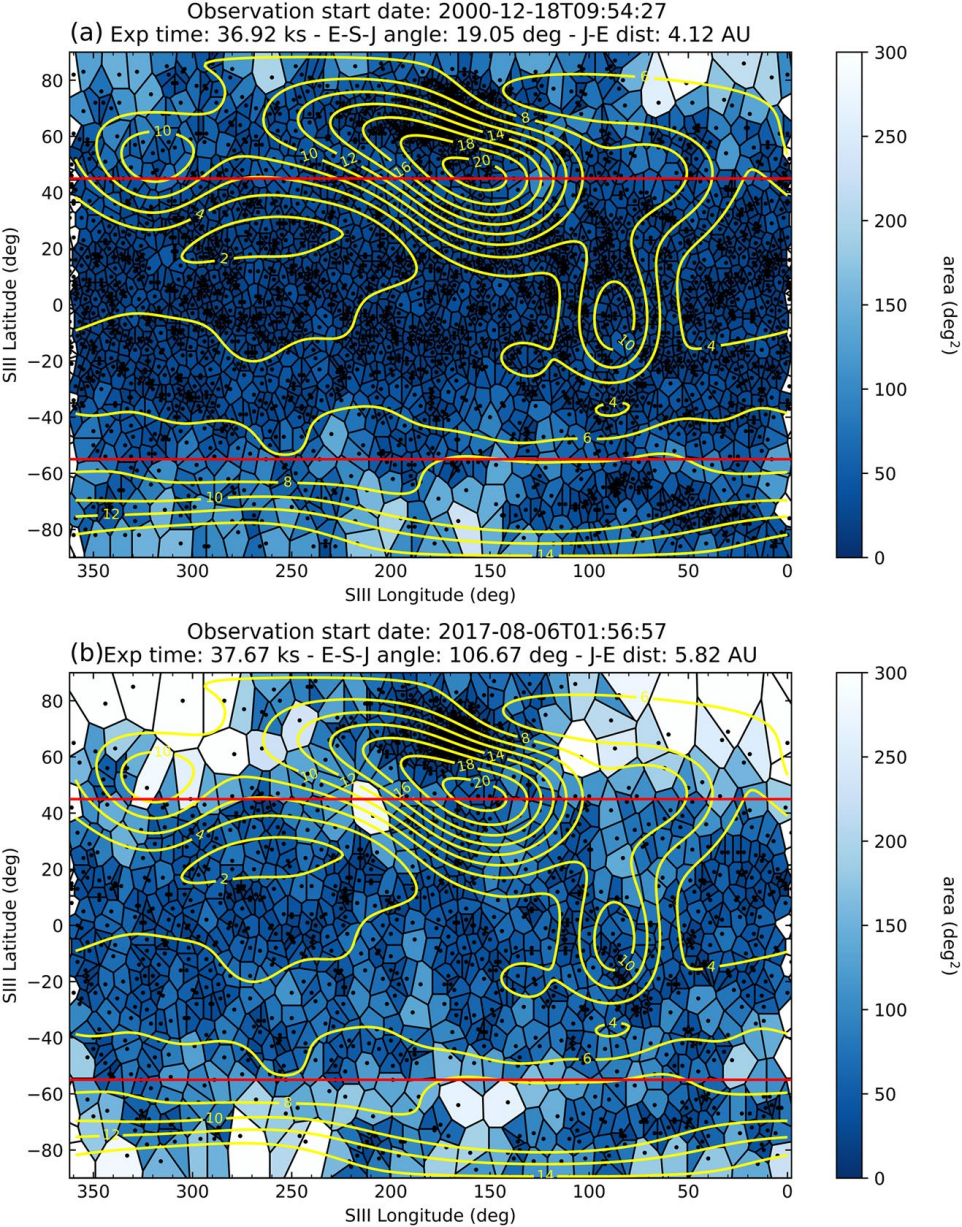
Voronoi tessellation diagrams with JRM09 internal magnetic field model overlaid (yellow contours) for observations coinciding with relatively high (a) (ObsID 1862)and low (b) solar activity (ObsID 20002). The borders of each polygon encompass the area around a photon which is closer to that photon than any other. The colourbar represents the area of the polygons (deg^2^). Clustering of photons will result in lower area polygons. Red horizontal lines represent the latitude boundaries of the disk region, which are 45° SIII latitude in the north and −55° SIII latitude in the south.

In addition to looking at the correlation between the Jovian disk X‐rays and solar X‐ray flux, the high spatial resolution of HRC allowed for the investigation of the spatial morphology of the disk photons. This analysis was conducted to explore the possibility of any spatial preference of X‐ray emission across the disk region. For this purpose, we employ Voronoi tessellation (VT) diagrams, based on the VOISE (VOronoi Image SEgmentation) algorithm (Guio & Achilleos, 2009), which were described in Section 2.2.4. Cases are shown in Figure 7 for observations coinciding with relatively high (a) and low (b) solar activity, using the same observations that were displayed in Figure 5.

In order to represent Jupiter’s internal magnetic field, we have incorporated the JRM09 (Juno Reference Model through Perijove 9) internal field model (Connerney et al., 2018), a spherical harmonic model of the magnetic field of Jupiter obtained from vector magnetic field observations taken by the Juno spacecraft during its first nine polar orbits of the planet. The surface magnetic field iso‐contours (yellow) were generated using the LesiaMag distribution (Cecconi et al., 2022), are overlaid on the tessellation plots in Figure 7, and aid in the identification of any spatial preference of the Jovian disk emissions. Also included are the latitudinal boundaries of the disk region (red) that were defined previously: 45° SIII latitude in the north and −55° SIII latitude in the south. Although auroral photons are included in the Voronoi tessellation diagrams in Figure 7, the planetary disk remains the key region of interest.

The result of the high‐solar flux case (panel a) is that uniformity is observed across the disk, with the majority of polygons having an area <50 deg^2^. Cases of higher solar activity are expected to reveal less of the underlying morphology of the disk as the expectation would be that stronger solar X‐ray flux would dominate the production and emission of Jovian disk X‐rays. Additionally, for observations that have an exposure time of roughly one full Jovian rotation (like these examples), each longitude would receive equal spatial coverage by the Sun. The result being that all longitudes will be illuminated by the Sun in equal proportion, making any spatial non‐uniformity of the disk X‐ray emission unlikely.

By contrast, instances of lower solar activity may allow more scope to search for distinct local anomalies in disk X‐ray morphology (Bhardwaj et al., 2005). Figure 7b represents a low solar activity case which allows us to search for a potential spatial preference to the emission. In this case, the Voronoi tessellation diagram is observed to have more variability than its high solar activity counterpart. A greater number of polygons are observed with areas >50 deg^2^, while we also see clustering of polygons of smaller areas.

It is difficult to identify any spatial clustering of Jovian disk photons in relation to surface magnetic field strength from the tessellation plots in Figure 7 alone, due to the fact that the iso‐contours differ greatly in terms of area, and also because many of the iso‐contours traverse the latitude constraints (defined in Figure 4) of the planetary disk region. It is evident that there is clearly a great deal of structure in the X‐ray clustering in both tessellation maps that is well above the statistical fluctuations and does not appear to be related to variations in the magnetic field. To extract more information from this analysis, we first split our data set into two groups: observations coinciding with (a) high and (b) low solar activity. For an observation to be considered to coincide with high solar activity, the corresponding peak GOES X‐ray light‐curve must exceed 10^−6^ Wm^−2^. As stated previously, this threshold was chosen because it represents the mean peak flux across the data set. A further constraint stated that the observation must encompass at least one full Jovian rotation. The result was that the high solar activity group contained six HRC‐I observations of Jupiter, while the low solar activity group contained 15 observations. Observations that fall into the high solar activity group are denoted “a” in Table 1, while low solar activity group observations display “b.” This distribution highlights the sparsity of HRC observations of Jupiter during periods of high solar activity, which can also be observed in the sunspot plot in Figure 1a. Of the 7 HRC observations that had an exposure time less than one full Jovian rotation, none coincided with a period of high solar activity.

**Figure 8:**
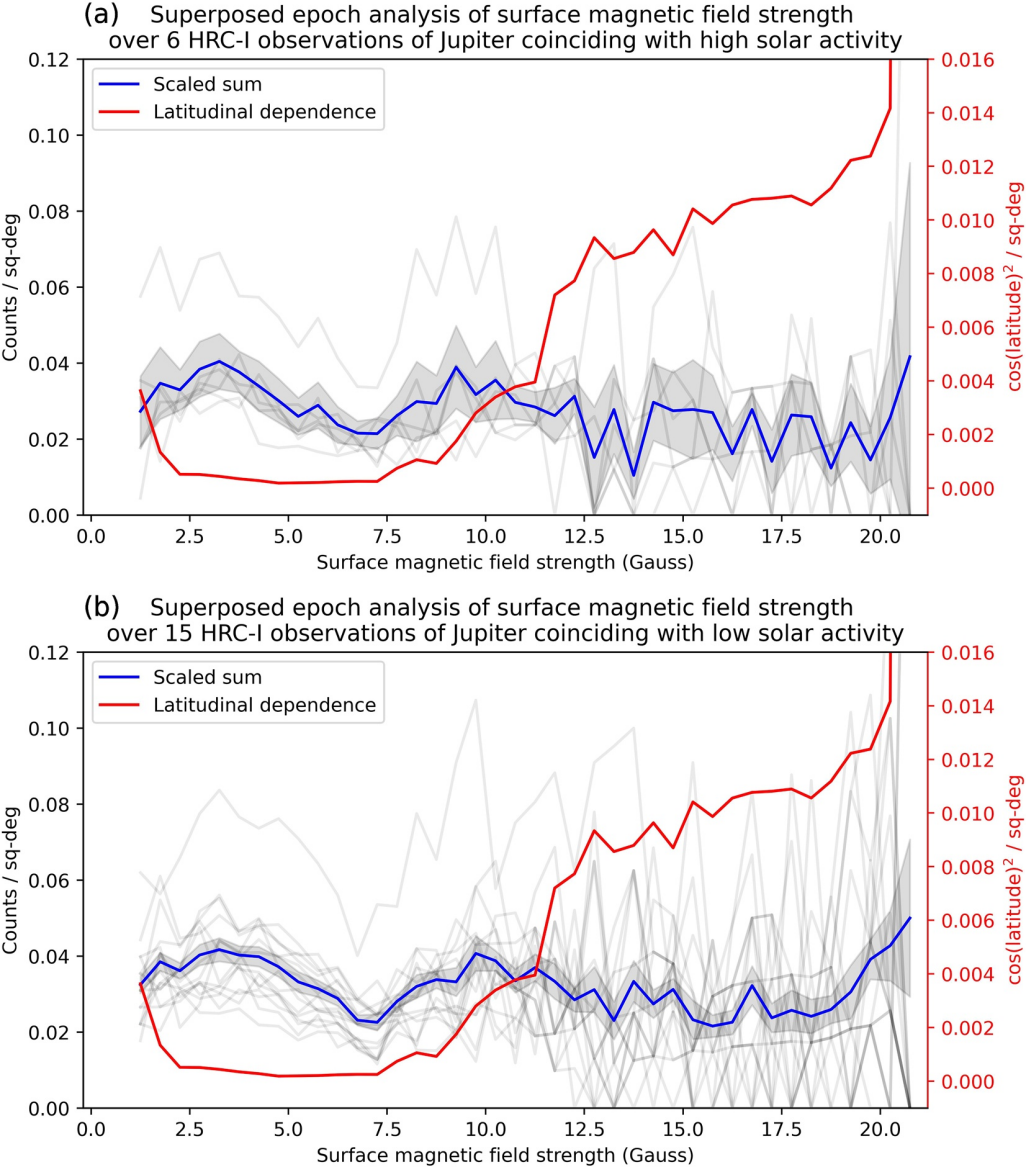
Histograms showing Jovian disk counts as a function of surface magnetic field strength (Gauss) for (a) six HRC observations of Jupiter coinciding with high solar activity and (b) 15 HRC observations coinciding with low solar activity. Data are presented in 0.5 G bins, and counts were normalized by the area of the planetary disk contained within each magnetic field bin (counts/sq‐deg). The scaled sum (blue), giving the mean counts/sq‐deg, is shown with 3*σ* error bars (shaded gray area). The latitudinal dependence (red) accounts for scattering across the curved surface of the planet using a cos(latitude)^2^ distribution, and is also normalized by area. This is included to examine how much of an effect the scattering angle has when observing the Jovian disk.

We then investigated the surface magnetic field strength at the location of each of the Jovian disk photons. The JRM09 model provides magnetic field data with 1° SIII longitude × 1° SIII latitude resolution. For each observation, a histogram is produced whereby the disk counts are binned into 0.5 Gauss bins (10,000 G = 1 T), and these counts are then normalized by the area of the disk that is contained within each of the magnetic field bins. The histograms are then combined in Figure 8 (gray lines) so that a superposed epoch analysis can be performed on both the high (panel a) and low (panel b) solar activity groups. The scaled sum (blue), which includes 3*σ* error bars (shaded gray area), was calculated by finding the mean counts/sq‐degree within each 0.5 G bin. Also included is a latitudinal dependence function (red) to account for scattering across the curved surface of the planet. Bhardwaj et al. (2006) found that the low‐to middle‐latitude Jovian X‐ray photons are consistent with the cosine‐squared dependence expected from a disk of uniform surface brightness. To examine how much of an effect the scattering angle has when observing the Jovian disk, we therefore apply a cos^2^ *θ* distribution over our disk region latitude range (−55°,+45°). This scattering distribution is also normalized by the disk area within each magnetic field bin. The sharp increase in the normalized latitudinal dependence at high surface magnetic field strengths is due to the fact that very few locations within the disk region have such high associated magnetic field strengths. Therefore, even if there are very few photons at these locations, the latitudinal dependence will be very high after accounting for the area.

In Figure 8, the scaled sum (blue) displays a general increase in mean counts/sq‐degree over the surface magnetic field strength range 2–3.5 G. This is observed for both the high (a) and low (b) solar activity groups, and is followed by a decrease in normalized counts over the range 4–7.5 G. Over this entire magnetic field range (2–7.5 G), the latitudinal dependence (red) remains flat, meaning that this increase and decrease in the normalized counts is not a latitudinal effect. This feature therefore appears to indicate the preference of some Jovian disk X‐ray emission to come from regions of lower magnetic field strength, and there is a sharp drop off when the magnetic field strength increases. This indicates the possible presence of another driver of a portion of the Jovian disk X‐ray emissions, and gives credence to the hypothesis put forward in Waite et al. (1997) and Gladstone et al. (1998) that a larger atmospheric loss cone in these regions of weaker surface magnetic field strength can lead to the precipitation of otherwise trapped electrons and ions from the radiation belts into the planet’s upper atmosphere, where they undergo charge exchange or bremsstrahlung interactions to produce X‐rays. Another interesting result is that this increase and decrease in the normalized counts appears for both the high (a) and low (b) solar activity groups, suggesting the possibility that radiation belt precipitation is still observable even in cases of high‐solar activity.

Numazawa et al. (2019, 2021) investigated emissions from the radiation belts using X‐ray data sets of Jupiter taken by the Suzaku X‐ray Imaging Spectrometer (XIS) (Mitsuda et al., 2007) in 2006 (Ezoe et al., 2010), 2012, and 2014. These observations revealed diffuse X‐ray emission in the 1–5 keV energy range associated with the Jovian inner radiation belts, and this diffuse emission remained observable at solar maximum in 2014 (Numazawa et al., 2019). Ezoe et al. (2010) suggested that inverse‐Compton scattering between ultra‐relativistic (tens of MeV) electrons in the radiation belts and visible solar photons was the most likely cause of this emission.

This study sets the foundation for a potential new avenue to use the Jovian X‐ray data sets to explore the radiation belts of Jupiter. Kollmann et al. (2021) presented Juno measurements suggesting that certain magnetic field regions close to the planet are not expected to be able to trap charged particles. If we consider the spatial analysis presented in this study (e.g., Figure 7), and use the latest magnetic field (JRM33, Connerney et al. (2022)) and current sheet (Connerney et al., 2020) models to map X‐ray photons on Jupiter’s surface to their source locations within the Jovian magnetosphere (using e.g., the JupiterMag package, James et al. (2022), or the LesiaMag distribution, Cecconi et al. (2022)), we can potentially locate and isolate the regions within the Jovian disk where radiation belt precipitation is most likely to occur. This could potentially benefit collaborative observing efforts in the future by enabling remote tracking of the loss of radiation belt particles into the Jovian atmosphere.

## 4 Summary

Here, we present a statistical study of the Jovian disk emissions using 19 years of Chandra HRC‐I data. We implement a Pulse Invariant filtering method to minimize background and ensure consistency across our data set in relation to instrument degradation over time. We compare the Chandra data to solar X‐ray flux data from the GOES XRS, resulting in a strong correlation between the two data sets, with a Pearson’s Correlation Coefficient of 0.9. Incorporating Voronoi tessellation diagrams, we identify a clustering effect that, on initial inspection, appears to be unrelated to variations in the surface magnetic field strength. However, after grouping cases of high and low solar activity, and comparing normalized counts to surface magnetic field strength, we find a preference for the disk emission in the 2–3.5 G region of surface magnetic field strength. This suggests that the production of the disk X‐ray emissions is predominantly governed by solar activity, but may also contain the imprint of radiation belt precipitation into the atmosphere.

## Data Availability

*NASA Chandra X‐ray Observatory* observations used in this study are available from the Chandra Data Archive http://cda.harvard.edu/chaser/. *NOAA’s GOES X‐ray Sensor* data used in this study can be found at https://www.ngdc.noaa.gov/stp/satellite/goes/index.html. The *online catalogue of the sunspot index* is available at http://www.sidc.be/sunspot-data/. Data analysis methods and code for this work are provided at https://github.com/SeanMcEntee/cxo_goes_disk_study. The data required to reproduce the results shown in this study are stored in the Zenodo repository: https://doi.org/10.5281/zenodo.7379645 (McEntee et al., 2022).
